# Intratumoral Virotherapy with Wild-Type Newcastle Disease Virus in Carcinoma Krebs-2 Cancer Model

**DOI:** 10.3390/v13040552

**Published:** 2021-03-25

**Authors:** Kseniya S. Yurchenko, Alexandra V. Glushchenko, Marina A. Gulyaeva, Yuhai Bi, Jianjun Chen, Weifeng Shi, Lyubov S. Adamenko, Alexander M. Shestopalov

**Affiliations:** 1 FRC of Fundamental and Translational Medicine, Eurasian Institute of Zoonotic Infections, Timakova Street 2, 630117 Novosibirsk, Russia; rimmaaltai2017@gmail.com (A.V.G.); mgulyaeva@gmail.com (M.A.G.); aminisib@yandex.ru (L.S.A.); shestopalov2@ngs.ru (A.M.S.); 2 Faculty of Natural Sciences, Novosibirsk State University, Pirogova Street 2, 630090 Novosibirsk, Russia; 3 CAS Key Laboratory of Pathogenic Microbiology and Immunology, Institute of Microbiology, Center for Influenza Research and Early-Warning (CASCIRE), Chinese Academy of Sciences, Beijing 100101, China; beeyh@im.ac.cn; 4 Wuhan Institute of Virology, Chinese Academy of Sciences, 44 Xiaohongshan, Wuhan 430071, China; chenjj@wh.iov.cn; 5 Key Laboratory of Etiology and Epidemiology of Emerging Infectious Diseases in Universities of Shandong, Shandong First Medical University & Shandong Academy of Medical Sciences, Taian 271016, China; shiwf@ioz.ac.cn

**Keywords:** Newcastle disease virus, virotherapy, oncolytic viruses, carcinoma Krebs-2, tumor progression

## Abstract

The results of experimental and clinical trials of the agents based on oncolytic Newcastle disease virus (NDV) strains provided hope for the development of virotherapy as a promising method for treating human tumors. However, the mechanism of the antitumor effect of NDV and realization of its cytotoxic potential in a cancer cell remains to be elucidated. In the current work, we have studied the antitumor effect of NDV in a syngeneic model of mouse Krebs-2 carcinoma treated with intratumoral injections of a wild-type strain NDV/Altai/pigeon/770/2011. Virological methods were used for preparation of a virus-containing sample. Colorimetric MTS assay was used to assess the viability of Krebs-2 tumor cells infected with a viral strain in vitro. In vivo virotherapy was performed in eight-week-old male BALB/c mice treated with serial intratumoral injections of NDV in an experimental model of Krebs-2 solid carcinoma. Changes in the tumor nodes of Krebs-2 carcinoma after virotherapy were visualized by MRI and immunohistological staining. Light microscopy examination, immunohistochemical and morphometric analyses have shown that intratumoral viral injections contribute to the inhibition of tumor growth, appearance of necrosis-like changes in the tumor tissue and the antiangiogenic effect of the virus. It has been established that a course of intratumoral virotherapy with NDV/Altai/pigeon/770/2011 strain in a mouse Krebs-2 carcinoma resulted in increased destructive changes in the tumor tissue, in the volume density of necrotic foci and numerical density of endothelial cells expressing CD34 and VEGFR. These results indicate that intratumoral NDV injection reduces tumor progression of an aggressive tumor.

## 1. Introduction

The widespread acceptance of the idea of using viruses as antitumor agents and encouraging results of successful experimental treatment of tumors obtained in numerous preclinical studies have led to the establishment and further development of targeted cancer therapy (virotherapy), which is based on the use of infectious oncolytic agents for the selective destruction of tumor cells. A wide range of wild-type and genetically modified viruses have been proposed as oncolytic agents [1,2]. One such candidate is the Newcastle disease virus (NDV), which belongs to the Avulavirus genus of the Paramyxoviridae family [3]. Oncolytic NDV strains isolated from pigeons are called pigeon paramyxovirus serotype 1 (PPMV-1) and present an antigenic variant of avian paramyxovirus serotype 1 (APMV-1) [4].

An important trend in the study of viruses with oncolytic potential is the identification of principally new strains allowing the use of wild-type non-pathogenic isolates as an antitumor agent. The use of animal viruses (or, as in the case of NDV, avian virus) that are non-pathogenic for humans in virotherapy can significantly reduce the risks of infectious diseases. Since NDV is an avian virus, it is not eliminated by the mammalian immune system due to the presence of the antibodies to the viral agent in the blood, while the existence of natural immunological response to the adenoviruses most commonly used in virotherapy, measles, herpes simplex, and mumps viruses limits their clinical use [5,6,7,8,9]. Moreover, wild-type viruses exhibit an antitumor effect and natural selectivity upon replication in cancer cells in the absence of any genetic manipulations. Despite the fact that the mechanism underlying the selectivity of the wild-type viruses for tumor cells is still under intense discussion, tumor cells are known to be the preferred substrate for virus replication, since they actively divide and have a mobilized synthesis machinery required for viral reproduction. Tumor tissue structure and microenvironment, which are characterized by changes in intercellular contacts, facilitate the adsorption of viral particles.

NDV requires neither additional development of the strategies for the effective viral delivery (since it displays a natural tumor tropism) nor overcoming the host antiviral immune response (since it belongs to the avian viruses).

NDV strains are divided into two groups based on their ability to infect human cells and replicate in them: lytic and non-lytic viruses. When studying both groups of NDV strains, several possible concepts for the use of their antitumor potential for the effective treatment of tumors have been proposed, which has led to the emergence of different virotherapy approaches. One of them is the use of lytic NDV strains for the selective destruction of malignant cells via the rapidly spreading cytotoxic effect on tumor cells due to effective viral replication and release of numerous infectious viral particles [10,11].

Most scientific papers that suggest possible mechanisms of virus-mediated tumor cell death mention apoptosis as the main specific pathway for manifestation of the antitumor cytotoxic potential in NDV-infected cells. While apoptotic cell death is rarely detected in a tumor of epithelial origin in in vivo experiments on antitumor therapy, in vitro apoptosis of cancer cells can be only caused by relatively high drug doses, which are high in terms of therapeutic doses for humans [12]. In recent years, there have been studies on the mechanism of virus-induced cancer cell lysis providing evidence that NDV is capable of triggering not only apoptosis but also autophagy [13] and necrosis/necroptosis [14,15] in malignant cells. Meanwhile, necrosis of solid tumors is quite often observed during cancer treatment in vivo, which is associated with direct activation of necrotic cell death mechanisms as well as with impaired supply of rapidly growing tumors with oxygen and nutrients.

In addition to the cytotoxic effect causing direct destruction of tumor cells, oncolytic viruses have also attracted researchers’ attention due to their antiangiogenic properties [16,17,18]. Newly formed vessels in the tumor tissue are believed to be susceptible to infection. Oncolytic viruses can infect and destroy tumor-associated endothelial cells [19] by indirectly stimulating the immune response as a reaction to vascular disruption [16] with the expression of viral proteins exhibiting antiangiogenic properties [20]. Viruses are known to be able to act on the blood vessels located in the tumor environment as well as affect the processes of intratumoral neoangiogenesis. Moreover, newly formed vessels in the tumor tissue are believed to be more susceptible to infection than those already existing in the tumor. It is considered that viruses infect epithelial cells (ECs) by entering them through the lumen-facing vessel wall surface. However, secondary infection of ECs through the basement membrane from the tumor tissue is also possible. Oncolytic viruses directly lyse intratumoral ECs, indirectly stimulate the immune response as a reaction to vascular disruption [16] and express viral proteins with antiangiogenic properties [20].

There are few experimental examples of the antiangiogenic properties of NDV. Nevertheless, a number of studies have shown that NDV strains can inhibit angiogenesis. An in vitro experiment in glioblastoma tumor cells demonstrated a significant antiangiogenic effect 18 h after infection of the cells with the Iraqi NDV strain as compared to the untreated control cells. Suppression of angiogenesis is observed upon inhibition of the expression of angiogenic factors such as fibroblast growth factor, keratinocyte growth factor, vascular endothelial growth factor, and inter-cellular adhesion volecule-1 [21,22]. Thus, the authors suggested that tumor remission in the tumor node can be not only due to the direct cytolytic effect of the virus but also via inhibition of the expression of angiogenesis factors, which induce blood vessel formation in the tumor.

Sui [23] and the group of researchers from China conducted an in vivo experiment to study the cytolytic activity of a NDV strain in human gastric carcinoma BGC-823 xenograft model and revealed the antitumor effect of NDV through inhibition of angiogenesis. Moreover, the angiostatic effect is assumed to be associated with a decrease in the number of CD31+ endothelial cells and inhibition of VEGF-A and MMP-2 in cancer cells.

In addition, it has been shown that hypoxia in NDV-infected tumor tissue, which often occurs during tumor development, enhances NDV-mediated oncolysis of human kidney cancer cells by a velogenic strain AF2240 isolated in Malaysia [24]. Furthermore, NDV infection is accompanied by the production of IFN-beta but not IFN-alpha by the tumor cells, which is important for successful virus replication. In addition, production of IFN-beta promotes cell death by apoptosis.

Another approach to virotherapy with NDV is based on the idea of a therapeutic antitumor effect via stimulation of non-specific immunity through the induction of cytokines and interferons, better recognition of tumor antigens by the immune system and stimulation of cytotoxic cells: natural killers and cytotoxic T lymphocytes [25,26].

The encouraging results obtained in preclinical and clinical trials on the antitumor effect of viruses dictate the need for further research on the virus-cell interaction mechanisms leading to the death of tumor cells.

In previously published studies, the NDV/Altai/pigeon/770/2011 strain [27] demonstrated a pronounced cytotoxic effect on human tumor cells in vitro and in a human lung carcinoma xenograft model [28]. The aim of this work was to study the antitumor effect of a NDV strain in a model of mouse Krebs-2 carcinoma treated with intratumoral injections of the NDV/Altai/pigeon/770/2011 strain.

## 2. Materials and Methods

### 2.1. Newcastle Disease Virus Strain and Tumor Cell Line

Newcastle disease virus NDV/Altai/pigeon/770/2011 strain [27] with an infectious titer of 7.2 ± 0.49 lgTCID50/mL ± 2σ at a tissue cytopathic dose on a Vero cell culture and hemagglutination inhibition titer in the reaction with rooster erythrocytes of 256 HAU per 100 µL was used in the study. The NDV strain was generated by inoculation into the allantoic cavity of chicken embryos on day 9 of embryonic development in accordance with standard WHO procedures [29]. Developing chick embryos were obtained from the State Research Center of Virology and Biotechnology Vector (Koltsovo, Novosibirsk Region, Russia). The virus-containing liquid was centrifuged at low speeds to remove coarse debris, passed through a 0.45 μm membrane filter and stored at −80 °C.

The presence of NDV in the allantoic fluid was determined by hemagglutination assay and real-time RT-PCR with specific primers. Viral RNA was isolated from the virus-containing liquid (VCL). Primary identification of the isolated Newcastle disease virus was performed by RT-PCR with real-time detection in a closed tube using primers and a probe specific to the conserved regions of the Newcastle disease virus M gene according to [30].

Experimental studies with Newcastle disease virus were carried out in accordance with the sanitary safety requirements for working with microorganisms of pathogenicity groups III–IV and helminths (SP 1.2.731, dated 25 January 2005).

An implantable strain of Krebs-2 ascites carcinoma was kindly provided by a senior scientist at the Laboratory of Gene Expression Regulation of the Institute of Cytology and Genetics, Popova N.A, PhD. Krebs-2 tumor cells were maintained through regular passages in the mouse. Having first occurred as a spontaneous breast tumor in a hybrid mouse, Krebs-2 was further converted to the ascitic form and, to date, Krebs-2 cells are presented with poorly differentiated forms. Krebs-2 cells do not contain genes of the major histocompatibility complex. Therefore, they are effectively transplanted into inbred mouse lines. A solid node is formed in the thigh region upon intramuscular implantation. The tumor does not give rise to metastases.

### 2.2. Evaluation of the Viability of Krebs-2 Ascites Carcinoma Cells Grown in Suspension Culture Infected with the Newcastle Disease Virus

To evaluate the cell viability after infection with Newcastle disease virus, suspension culture of mouse Krebs-2 ascites carcinoma was infected with a NDV strain. For this, a suspension of Krebs-2 cells in DMEM medium was aliquoted into Eppendorf microtubes. Cells were concentrated by centrifugation at 1500 g for 5 min, the supernatant was removed, and the cells were incubated with different dilutions of the virus: 2, 8 and 16 HAU per 10,000 cells. Virus dilutions were prepared in DMEM medium (Gibco Inc.) containing 10% FBS (Gibco Inc.) and 60 μg/mL gentamicin sulfate. Cells were incubated with the virus for 1 h 30 min at 37 °C in a 5% CO_2_ incubator. Next, the cells were concentrated by centrifugation at 1500 g for 5 min, virus-containing supernatant was removed, and the cell pellet was resuspended in fresh growth medium and seeded in a 96-well plate at a concentration of 10,000 cells per well in a volume of 100 μL. Control untreated cells were incubated in growth medium under the same conditions.

Cell viability was measured by colorimetric assay using CellTiter 96 AQueous One Solution Cell Proliferation Assay kit (Promega) according to the manufacturer’s protocol four days after infection with NDV strains. The reagent contains a tetrazolium compound, MTS, which is reduced to formazan and stains intact cells with enzymatic activity. A total of 20 μL of the reagent was added to each well containing 100 μL of culture medium; cells were incubated for 2 h at 37 °C in the presence of 5% CO_2_. The optical density was measured at a wavelength of 490 nm on an Elx808 Absorbance Microplate Reader (Biotek).

Viability of the control Krebs-2 carcinoma cells was evaluated 4 days after infection with NDV strains based on the percentage of living cells versus the untreated control.

### 2.3. Study of the Antitumor Effect of Virotherapy in Vivo

Animals were fed a standard diet with free access to food and water. The adaptation period was one week prior to the onset of experiments. The study was carried out in compliance with the principles of humanity described in the Guidelines of the European Community (86/609/EEC), Helsinki Declaration and in accordance with the Order No. 755 of the USSR Ministry of Health on 12.08.1977 “On measures for further improvement of organizational forms of work using experimental animals”. Viral experiment consisted of two stages.

At the first stage, a pilot experiment was performed using 4 eight-week-old male -BALB/c mice. Krebs-2 ascites carcinoma was implanted intramuscularly into the thigh of all mice at a concentration of 500,000 cells in 100 μL of PBS. On the third day after implantation, two experimental mice were injected intratumorally in the thigh with 100 μL of the NDV/Altai/pigeon/770/2011 strain at the maximum concentration (256 HAU per 100 μL), and two control mice received physiological saline. Single injections were administered daily for 5 days. On day 28 after tumor implantation, the spread of the intramuscular tumor process was assessed by magnetic resonance imaging (MRI) at the Center for Genetic Resources of Laboratory Animals of the SPF Vivarium at the Institute of Cytology and Genetics. After MRI, animals were sacrificed by dislocation of the cervical vertebrae, solid tumor node with the surrounding muscle tissues were collected for further morphological examination by light microscopy.

To study the dynamics of tumor progression and pathomorphological changes in Krebs-2 carcinoma tissue treated with intratumoral injections of the NDV/Altai/pigeon/770/2011 strain, the second stage of the in vivo experiment was carried out. The study was conducted in 60 7-to-8-week-old white male BALB/c mice obtained from the mouse bank of the State Research Center of Virology and Biotechnology Vector (Koltsovo, Russia). A total of three groups of animals, each consisting of 20 mice, were formed. Krebs-2 ascites carcinoma was implanted intramuscularly into the thigh of all mice at a concentration of 500,000 cells in 100 μL of PBS. The first control group was comprised of animals not receiving injections after tumor implantation. The second, control (allantois), group consisted of animals receiving injections of the uninfected (virus-free) allantoic fluid of the chick embryo. The third, treated, group included animals that received a five-day course of intratumoral virotherapy starting from the third day after tumor implantation at a dose of 100 μL of the NDV/Altai/pigeon/770/2011 strain containing 256 HAU per 100 µL.

Viral injection dose, the scheme of virotherapy and allantoic fluid administration, the volume of injections, and housing conditions were completely similar to those of the first experiment. Animals were weighed on a daily basis. Tumor growth was daily assessed by measuring the affected limb using a caliper. The tumor volume (mm3) value was obtained by multiplying three orthogonal measurements. On day 5, 10, 15, and 20 after completion of the injection course (day 13, 18, 23, and 28 of tumor growth, respectively), 5 animals of each group were sacrificed at a time by dislocation of the cervical vertebrae; tissue samples were collected similarly to the first experiment.

### 2.4. Histological Examination

For light-optical examination, a Krebs-2 tumor tissue sample isolated from the mouse thigh muscle was fixed in 10% formalin at +4 °C for three days. Next, according to a standard protocol, the samples were dehydrated in a series of graded concentrations of alcohol, then butanol and xylene and embedded in HISTAMIX paraffin. The obtained paraffin blocks were divided into 4.5–5-μm-thick sections on a Microm HM355S rotary microtome (Thermo Fisher Scientific). Deparaffined sections were subjected to hematoxylin-eosin overview staining according to the standard procedure and embedded in Vitrogel mounting medium. The stained samples were examined on an Axio Imager A1 microscope with an AxioCamMRc camera using AxioVision software (rel. 4.12) (ZEISS) at the “Modern Optical Systems” core facility of the Federal Research Center of Fundamental and Translational Medicine.

### 2.5. Immunohistochemical Study

Polyclonal antibodies against the whole virion of Newcastle disease virus were generated by twice immunization of rabbits with the NDV/Altai/pigeon/770/2011 strain in complete Freund’s adjuvant with a 23-day interval. On day 46, blood serum samples treated with receptor-destroying enzyme (RDE) were obtained and analyzed for the presence of specific antibodies. The IgG fraction of rabbit antiserum samples was purified by affinity chromatography on a protein A-Sepharose 4B column in 0.02 M potassium phosphate buffer (pH 7.3), 0.15 M NaCl, 0.02% sodium azide at the laboratory of Biotechnology of the Institute of Chemical Biology and Fundamental Medicine (Novosibirsk, Russia).

For virus detection, tumor node sections were dewaxed and rehydrated, antigens were unmasked in citrate buffer (pH 9.0) in a microwave oven at 700 W. Sections were incubated with primary antibodies at 37 °C for 30 min, then with streptavidin–peroxidase complex, NovoLink DAB substrate buffer (Novocastra) and additionally stained with Mayer’s hematoxylin.

Krebs-2 tumor samples were also stained by immunohistochemistry [31] with primary antibodies for CD34 (Abcam) and VEGFR (Novocastra). NovoLink detection system (Novocastra) was used for visualization; sections were additionally stained with Mayer’s hematoxylin.

Histological samples were analyzed on an Axio Imager A1 microscope with an AxioCamMRc camera using AxioVision software (rel. 4.12) (ZEISS). Morphometric analysis of the tissue structural elements was conducted using a closed test system consisting of 100 testing points in a testing area equal to 3.64 × 10^5^ μm2; 50–100 visual fields were counted. Morphometric study included evaluation of the degree of structural changes in the tumor tissue taking into account the volume density of necrosis (Vv), numerical density of the microvasculature vessels (Nai) and endothelial cells expressing the selected IHC markers.

### 2.6. Statistical Data Processing

Mean values of the parameters for the assessment of Krebs-2 cell viability were determined using the standard STATISTICA V.6.0 software package; Student’s t-test was used. The differences were considered significant at *p* < 0.05. Cell viability assessment results are presented on a histogram as relative mean values of the percentage of living cells on the fourth day after treatment with virus to the percentage of control untreated cells plus standard deviation (mean relative value ± standard deviation).

## 3. Results

### 3.1. In Vitro Eevaluation of the Viability of Mouse Krebs-2 Ascites Carcinoma

**Figure 1:**
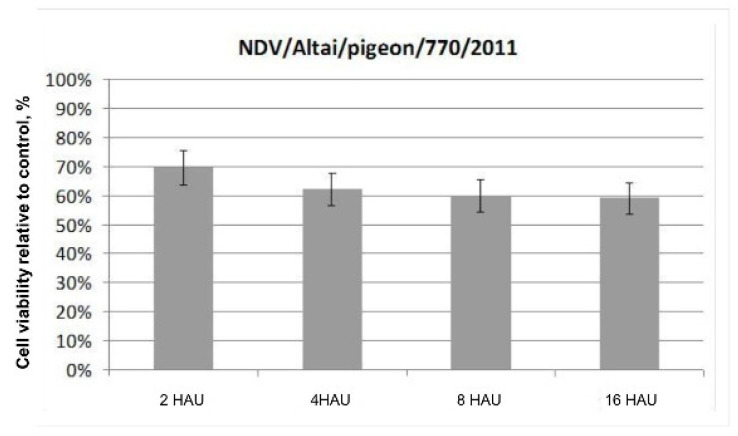
Cytotoxic effect of the wild-type NDV strain NDV/Altai/pigeon/770/2011 on mouse Krebs-2 ascites carcinoma cells on day 4 after infection, doses of 2, 4, 8, and 16 HAU per 10,000 cells, MTS assay.

The results of the colorimetric MTS assay presented as a histogram in Figure 1 demonstrate the cytotoxic effect of the wild-type NDV/Altai/pigeon/770/2011 strain on Krebs-2 carcinoma cells in vitro on day 4 after infection. No significant differences in the dose-dependent effect of the oncolytic action of the wild-type NDV strain were noted when the infectious virus dose was increased from 2 to 16 HAU per 10,000 cells. The destructive effect of the NDV/Altai/pigeon/770/2011 strain on Krebs-2 carcinoma contributes to a 69.5 ± 5.97% decrease in the viability of tumor cells after infection with the strain at a dose of 2 HAU per 10,000 cells; when the dose is increased to 16 HAU per 10,000 cells, cell viability decreases by another 10% to the value of 59.00 ± 5.60%.

### 3.2. Pilot In Vivo Experiment on BALB/c Mice

In a pilot experiment, Krebs-2 tumor cells were implanted intramuscularly in the right thigh of all four mice. On the day 3 after implantation, a palpable mass was observed, which is a characteristic of tumor formation. NDV/Altai/pigeon/770/2011 virus strain was injected intratumorally in the experimental group of mice starting from the onset of tumor formation (day 4 after tumor implantation). For this purpose, 100 μL of VCL was injected into the tumor node site in the experimental animals at a concentration of 256 HAU per 100 μL. No specific conclusions regarding changes in the tumor size, its progression or the onset of regression can be drawn during the next five days of the experiment, in which intramuscular injections of the virus were performed, since the inflammatory process develops in the tumor area in the first stages of virus introduction, which causes swelling and hinders visual assessment of possible changes.

Tumors implanted in control animals were treated with saline injections according to a similar scheme and at the same doses as for the experimental group. Similar processes (onset of the inflammatory process, tissue swelling) were observed in the control group receiving intratumoral injections of saline.

On day 10 after tumor cell implantation, the tumor-bearing thigh of the control mice was visually larger in size due to tumor growth than in experimental mice receiving virotherapy with a NDV strain. During the experiment, mice of the both groups actively consumed food and water. However, control animals not receiving virotherapy became less active by day 14 of the experiment.

**Figure 2:**
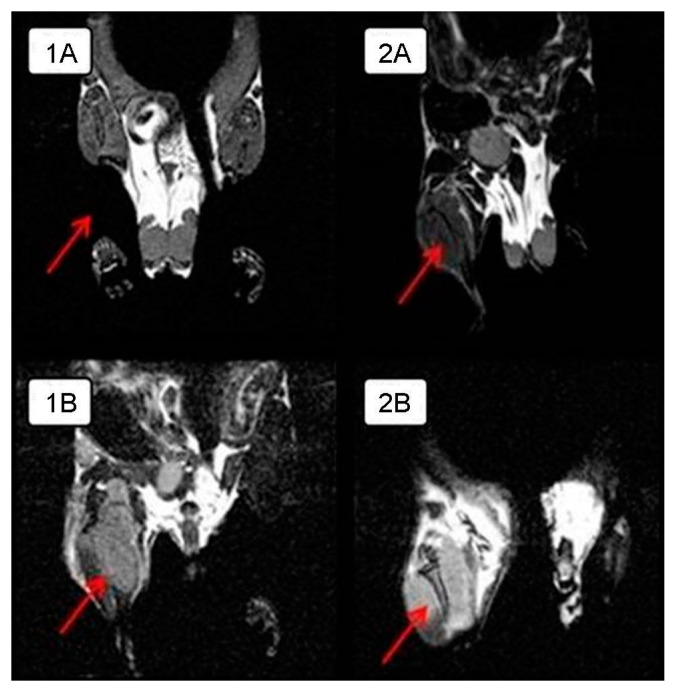
MRI of intramuscular Krebs-2 solid tumor nodes in BALB/c mice in the frontal plane on day 28 from the onset of the experiment. Arrows indicate the tumor. A. Experimental mice receiving intratumoral injections of NDV/Altai/pigeon/770/2011 at a dose of 256 HAU per 100 μL for 5 days: (**1A**)—complete tumor regression compared to the control animals, (**2A**)—significant tumor reduction compared to the control animals; (**1B**,**2B**)—control mice treated with saline injections.

In order to visualize the tumor node growth in detail in the control group and compare the differences in the size of the intramuscular tumor with those of the experimental group animals, MRI was performed on day 28 of the experiment. The tumor is clearly visualized next to the normal tissues in a series of frontal sections on MRI. The obtained MRI data revealed almost complete tumor regression in one experimental mouse and a significant tumor reduction in another mouse compared to the control animals (Figure 2); intramuscular thigh tumor is clearly visualized next to the normal tissues.

### 3.3. Pathomorphological Features of Krebs-2 Carcinoma after Intratumoral Virotherapy In Vivo (Pilot In Vivo Experiment)

**Figure 3:**
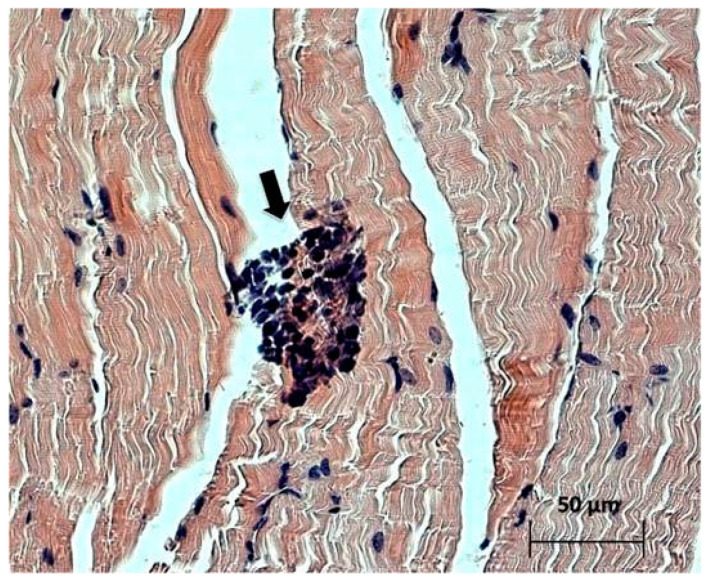
Individual tumor islets (indicated by an arrow) of Krebs-2 carcinoma in the muscle tissue of a mouse receiving a series of intratumoral NDV/Altai/pigeon/770/2011 injections with almost complete tumor regression in MRI. Day 20 after virotherapy (day 28 of tumor growth), hematoxylin-eosin staining.

**Figure 4:**
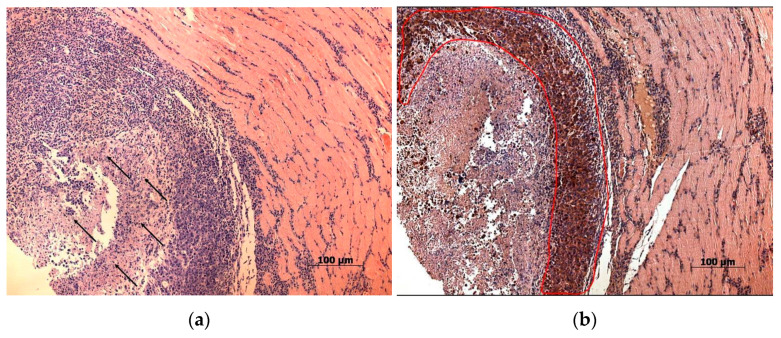
(**a**) Necrotic changes in a Krebs-2 solid tumor node of a mouse receiving NDV/Altai/pigeon/770/2011 injections and with a significant tumor reduction in MRI. Day 20 after virotherapy (day 28 of tumor growth), hematoxylin-eosin staining, 10× magnification. Arrows indicate necrotic areas in the tumor node; (**b**) Krebs-2 solid tumor node, immunohistochemical evaluation of the NDV/Altai/pigeon/770/2011 strain. Day 20 after virotherapy (day 28 of tumor growth), additional staining with hematoxylin, 10× magnification. Tumor cells showing a positive reaction with antibodies against the whole virion of NDV are encircled in red.

Study of the paraffin sections of tumor nodes obtained from experimental animals receiving intratumoral injections of the NDV/Altai/pigeon/770/2011 strain confirmed the almost complete absence of a tumor node (with the exception of individual tumor islets) in one mouse (Figure 3). Necrotic processes are observed in Krebs-2 carcinoma node in the sections obtained for another mouse (Figure 4a). Immunohistochemical analysis (Figure 4b) confirmed the presence of the NDV/Altai/pigeon/770/2011 strain in Krebs-2 carcinoma cells after intratumoral virotherapy. Virus-infected tumor cells were located mainly in the periphery of the necrotic site in the tumor node. These results suggest the cytotoxic effect of the virus in Krebs-2 cells at the site of intratumoral virus inoculation, which causes necrotic death in the central part of the tumor node, as well as viral replication and the spread of new viral progeny to the neighboring tumor cells from the center to the periphery of the tumor node.

**Figure 5:**
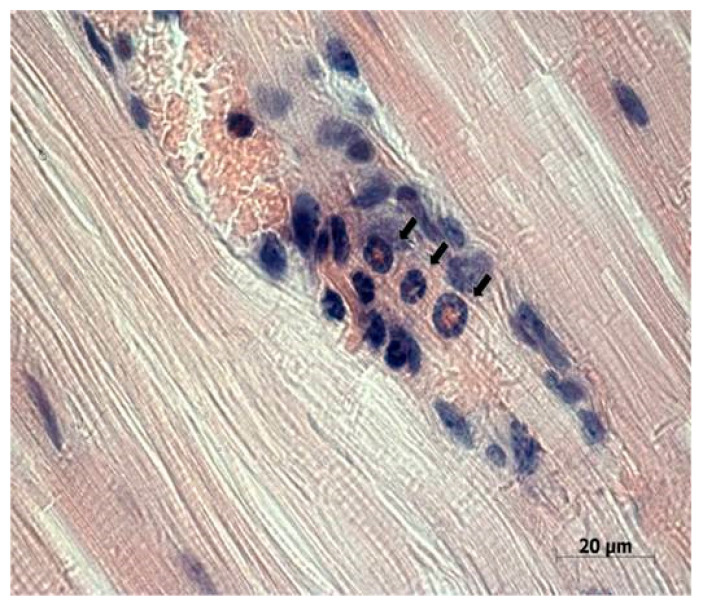
Neutrophils, lymphocytes, and macrophages (denoted by arrows) in the muscle tissue adjacent to the Krebs-2 tumor node in a mouse receiving a serious of NDV/Altai/pigeon/770/2011 strain injections and with a significant tumor reduction in MRI. Day 20 after virotherapy (day 28 of tumor growth), hematoxylin-eosin staining.

Cells of the immune system, such as neutrophils, lymphocytes, and macrophages, were detected in the muscle tissue adjacent to the tumor node (Figure 5), which indicates possible existence of some mediated immunostimulatory effect of the virus in addition to its direct cytotoxic effect.

### 3.4. Study of the Antitumor Activity of the Wild-Type NDV/Altai/pigeon/770/2011 Strain on the Progression of a Solid Tumor Node of Mouse Krebs-2 Carcinoma in BALB/c Mice In Vivo

A solid node was visually noticeable at the site of tumor cell implantation on day 7 of the experiment. Tumor growth and progression were observed in the control groups, while a decrease in the tumor growth rate was noted in the group receiving virotherapy.

Macroscopic examination showed that the tumor node in the thigh has a round-oval shape with clear boundaries with the surrounding tissue, hemorrhages and necrosis foci appear with an increase in the tumor volume. Light optical microscopy revealed that the central tumor region is represented by broad areas of necrosis containing islets of large tumor cells of various sizes. The subcapsular tumor area presents dense tissue consisting of poorly differentiated cells with large nuclei, in which numerous impaired mitotic events are observed.

**Figure 6:**
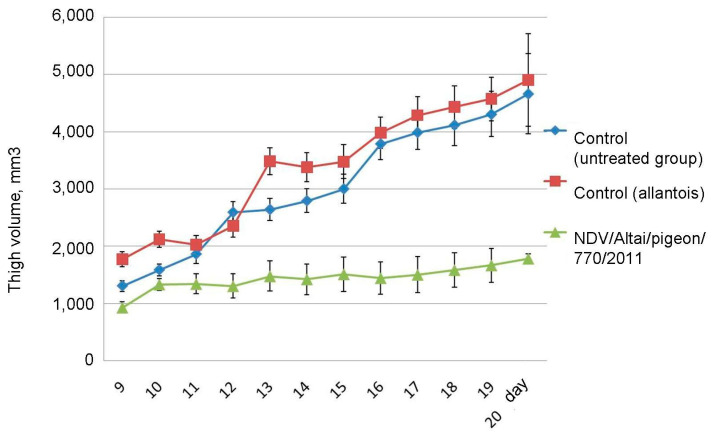
Changes in the intramuscular Krebs-2 tumor-bearing thigh volume in untreated groups and animals receiving injections of live Newcastle disease virus. Control—group not receiving injections; Control (allantois)—group receiving injections of uninfected chick allantois; NDV/Altai/pigeon/770/2011—group receiving injections of wild-type NDV strain obtained from the allantoic fluid of embryonated chicken eggs. Day—time after the end of the injection course. Results are presented as relative mean values ± standard deviation.

On day 20 after the end of the series of injections, the average size of the tumor-bearing thigh in the group of mice receiving live virus injections was 1779.1 ± 79.98 mm3, which is 2.6 times lower than that of the control group and the group receiving injections of uninfected chick allantois: 4659.9 ± 704.75 mm3 and 4899.1 ± 807.69 mm3, respectively (Figure 6). Tumor development and progression were observed in the groups of untreated mice, while inhibition of tumor growth was noted in animals receiving virotherapy.

### 3.5. Comparative Analysis of the Tumors Obtained from Untreated Animals and Animals Receiving Virotherapy Using Histological, Immunohistological, and Morphometric Methods

The microscopic specimen was tumor tissue consisting of atypical round tumor cells; a large amount of necrotic cells were noted. Muscle fibers destroyed due to invasive tumor growth were observed on the microscopic slides. No metastasis was observed.

**Figure 7:**
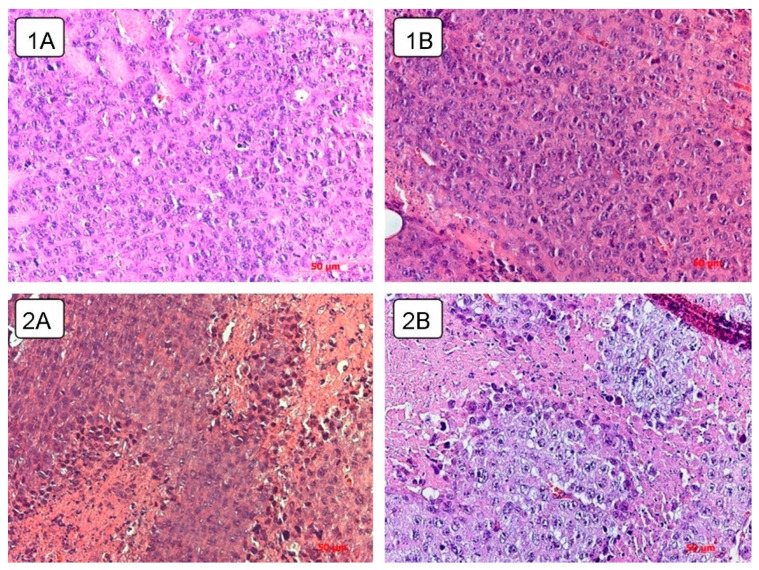
Necrotic changes in a Krebs-2 solid tumor node. 1A, B—necrotic areas in the tumor tissue of mice without virotherapy on (**1A**) day 5 (day 13 of tumor growth), (**1B**) day 20 (day 28 of tumor growth), hematoxylin-eosin staining, 40x magnification. 2A, B—necrotic areas in the tumor tissue of mice on (**2A**) day 5 after virotherapy (day 13 of tumor growth), (**2B**) day 20 after virotherapy (day 28 of tumor growth), hematoxylin-eosin staining, 40× magnification.

Spread of the necrotic area was observed in the tumor tissue of the control untreated and experimental animals receiving live virus (Figure 7). A total of 8.8 ± 1.76% of the volume density of necrosis was found in the control animals on day 5 after the course of injections of physiological saline. By day 10 and 15, the proportion of necrosis was 13.5 ± 1.34% and 15.3 ± 2.51%, respectively, and, by the 20th day, it increased to 19.8 ± 3.55% (Table 1).

Live virus injections triggered necrotic processes on day 5, which occupied 16.7 ± 2.35% of the tissue volume density. This parameter slightly changed by the day 10 and 15 and estimated 15.5 ± 1.51% and 13.6 ± 1.95%, respectively. On the day 10 and 15, the value of the volume density of necrosis corresponded to that of the control group. However, by the 20th day, the value increased to 25.7 ± 2.68%.

As a result, an increase in the volume density of necrosis is observed in the tumor node in all groups of animals. However, in the control group, the growth rate was the slowest with a maximum of 19.8 ± 3.55%, while intratumoral injection of the wild-type NDV/Altai/pigeon/770/2011 strain by the day 5 contributes to the high necrosis density value, which increases significantly (to 25.7 ± 2.68%) by the day 20.

Numerical density of blood vessels in the tumor tissue was found to be approximately 2 or more times lower after virus injection than in the control group. Morphometric analysis of large blood vessels (Table 1) in the tumor of control animals showed that the number of large visually distinguishable vessels in the tumor tissue sharply decreases by 4-fold on the day 10 compared to the day 5. This value remains unchanged on day 15. By the day 20, the number of large vessels in the field of view increases on average more than 4-fold. As early as on the day 5, the average number of large vessels is two times lower in animals receiving virotherapy than in the control group. A slight decrease in the number of vessels, which is similar to that in the control, is observed in the experimental group on days 10 and 15. However, on the 20th day, the number of vessels in experimental animals remains insignificant, while a sharp increase is observed in the control group.

In order to more accurately estimate the number of vessels and study the mechanism of neoangiogenesis inhibition, the numerical density of endothelial cells expressing hematopoietic marker CD34 and marker VEGFR was determined. Expression of these markers was observed in the tumor cells of control and treated mice, with a tendency of the number of cells immunopositive for CD34 and VEGFR to decrease in the treated group.

**Figure 8:**
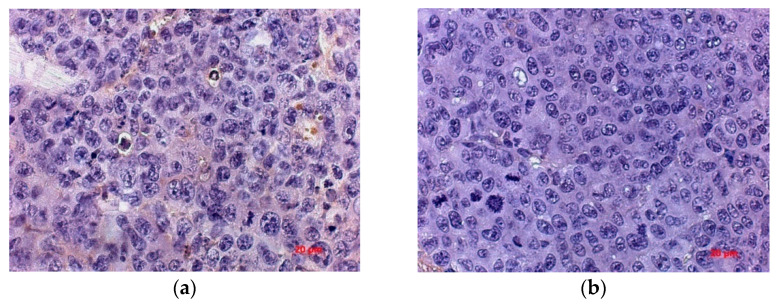
Immunohistochemical assay with the CD34 marker in endothelial cells in mice tumor tissue without virotherapy (**a**) and after a course of virotherapy (**b**) on the 20th day after the course of injections (28th day of tumor growth), 63× magnification.

The numerical density of CD34+ cells in the tumor of the treated group was lower by 72.69% and by 57.57% than in untreated mice by the day 5 and 20 after virotherapy, respectively. By day 20 after virotherapy, the number of CD34+ cells was 2.36 times lower in the tumor of mice treated with the virus than in untreated animals, with a 62.3% difference in the parameter from that of the treated animals by day 5 (Figure 8).

**Figure 9:**
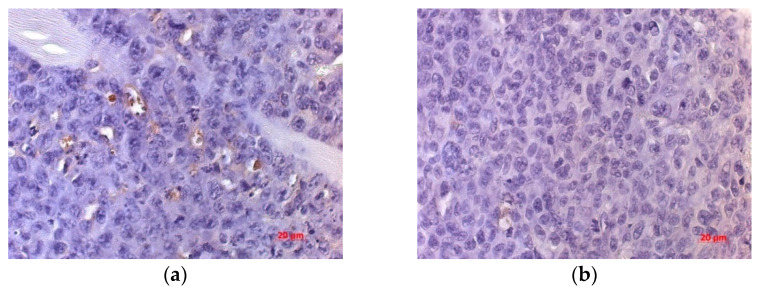
Immunohistochemical assay with the VEGFR marker in endothelial cells in mice tumor tissue without virotherapy (**a**) and after a course of virotherapy (**b**) on the 20th day after the course of injections (28th day of tumor growth), 63× magnification.

An IHC study of VEGFR expression in the tumor of mice inoculated with NDV/Altai/pigeon/770/2011 showed a significant decrease in the numerical density of VEGFR-expressing cells (by 37.45%) compared to that for the non-injected group on days 10 and 20 of observation (Figure 9).

## 4. Discussion

Oncolytic viruses as agents for antitumor therapy offer several important advantages over traditional cancer treatment approaches and other targeted therapies: (1) tumor-selective infection and viral reproduction in the tumor cell, leading to death, without cytotoxic effects on healthy cells; (2) involvement of infected tumor cells in cytolytic death by direct membrane lysis, in addition to activation of cell death mechanisms; (3) viral reproduction by replication in cancer cells, ensuring the spread of infectious particles in the tumor tissue after the release of the viral offspring, which increases the number of active agent and the number of infected cells and, as a result, increases the therapeutic effect; (4) the death of tumor cells (by direct oncolysis or programmed cell death) increases the pro-inflammatory microenvironment of the tumor and the involvement of immune system cells.

With all of these broad therapeutic options, tumor NDV-therapy has great potential for cancer treatment [32], but for the successful use of virotherapy, viruses must meet strict safety and efficacy criteria and carry out together with complex combined treatment.

Studies conducted in an animal model of experimental oncogenesis were aimed at investigating the effect of the course of virotherapy injections of the wild-type strain NDV/Altai/pigeon/770/2011 on the progression of mouse Krebs-2 carcinoma in vivo. The animal model and the method of virus introduction were selected in a pilot experiment on virotherapy of mouse Krebs-2 carcinoma. To confirm the oncolytic potential of Newcastle disease virus, the viability of mouse Krebs-2 tumor cells was first assessed in an in vitro system using the selected mesogenic strain NDV/Altai/pigeon/770/2011.

The in vitro study result demonstrates that the NDV/Altai/pigeon/770/2011 strain can be used for further works on experimental virotherapy in an animal model despite the fact that the oncolytic potential in relation to the mouse Krebs-2 carcinoma cells is significantly lower than that obtained in a similar experiment using human tumor cell lines [28].

Pilot experiment was performed on a small number of animals. The aim of the study was to assess intramuscular oncogenesis of the Krebs-2 carcinoma with the formation of a solid tumor node in an in vivo model, to monitor the condition of animals during tumor progression and to preliminary estimate the antitumor effect of a series of intratumoral injections of the wild-type strain NDV/Altai/pigeon/770/2011 of the Newcastle disease virus.

The second stage of the experiment on the model of mouse Krebs-2 carcinoma was aimed at the study and assessment of changes in the development of a solid tumor node at different time points after a course of intratumoral virotherapy and evaluation of morphological changes in the tumor tissue after virus injection in comparison with untreated animal tumors.

The obtained data on the tumor growth inhibition after serial intratumoral injections of Newcastle disease virus required clarification of the morphological changes and possible mechanisms of tumor growth suppression.

Necrotic foci are observed in the experimental group at different time points as compared to the tumor tissue of control animals and animals receiving a course of virotherapy on day 5 and day 20 (Figure 7). Intratumoral inoculation of the Newcastle disease virus strain NDV/Altai/pigeon/770/2011 contributes to the earlier and more widespread necrotic changes.

Assessment of the immune status after a course of virotherapy is complicated, since no cells involved in immune response activation (neutrophils, leukocytes, and macrophages) were found in the tumor tissue of experimental animals. Presumably, this may indicate a predominantly direct effect of the virus on the tumor tissue via direct oncolysis of tumor cells with the appearance of extensive necrotic foci.

It is possible that the appearance of extensive necrotic lesions in the control group may reflect the formation of ischemic foci in the tumor tissue resulting from the rapid tumor node growth and delayed neoangiogenesis.

We suggest that extensive necrotic lesions in the control group may reflect the formation of ischemic foci in the tumor tissue resulting from the rapid tumor node growth.

There is a correlation between an increase in the tumor node volume and an increase in the volume density of necrosis in the tumor tissue, which is due to the rapid tumor growth and the lack of feeding vessels. However, it should be noted that virus injections contribute to necrotic changes in the earlier stages of tumor growth and the appearance of a significant proportion of necrosis at a later date.

A sharp decrease in the number of large visually distinguishable vessels in the tumor tissue of the control animals on day 10 is, apparently, due to the high tumor growth rates, when new vessels do not have time to form and, as a result, nutrient supply of the tumor tissue is disturbed. On the contrary, an increase in the number of vessels by day 20 indicates that neoangiogenesis takes place in the tumor tissue.

There is almost a 5-fold difference in the number of large vessels between the control and experimental groups on day 20 of the experiment. Apparently, a small number of vessels in the experimental group on the 20th day indicates that virus is able to directly or indirectly affect neoangiogenesis of the growing tumor by regulating nutrient supply to the tissue.

Based on the estimation of numerical density of endothelial cells expressing CD34 in Krebs-2 carcinoma tissue, we suggest that intratumoral injection of the strain NDV/Altai/pigeon/770/2011 indicates that virus is able to directly or indirectly affect neoangiogenesis of the developing tumor, interfere with normal regulation of the nutrient supply to the tumor tissue and result in extensive necrotic lesions.

In order to explore the mechanisms of neoangiogenesis inhibition, an IHC study was carried out using a marker to the VEGF receptor, which is involved in the signal pathways that serve as a target for the majority of drugs inhibiting angiogenesis.

It is possible that the virus facilitates suppression of the angiogenic signal by decreasing the expression of endothelial factors and suppressing vascularization after viral therapy. We suggest that one of the mechanisms responsible for the delayed processes of neoangiogenesis in the tumor tissue may be the suppression of VEGF receptors.

Thus, the conducted study demonstrated that intratumoral injections of the wild-type NDV/Altai/pigeon/770/2011 strain in BALB/c mice with intramuscular Krebs-2 carcinoma had a restraining effect on the tumor node growth in comparison with the untreated animals. There is no doubt that virus-induced necrotic changes in the tumor serve as the main mechanism for the Krebs-2 carcinoma cell death. Apparently, the effect of intratumoral injections of the NDV/Altai/pigeon/770/2011 strain on tumor angiogenesis is an additional mechanism suppressing tumor growth, which affects regulation of the nutrient supply to the tumor tissue and, as a result, leads to the spread of extensive necrotic foci.

Apparently, the Newcastle disease virus circulating in a population of wild birds is a promising object for the analysis on the presence of antitumor properties. NDV isolated from a natural reservoir has been shown to be able to successfully kill tumor cells with various etiology and histogenesis. Modern research methods have made it possible not only to determine the effectiveness of the oncolytic effect of NDV strains but also study the mechanism of virus-mediated cell death, explore the interaction between the virus and the tumor cell and analyze the effect of the virus on the features of the immune response activation.

Furthermore, the obtained results indicate that wild-type NDV strains can serve as the basis for creation of an effective antitumor drug. The cytotoxic action of the NDV strains on the tumor cell lines and safety for the healthy cells demonstrates their effectiveness in the fight against cancer. Study of the antitumor activity of NDV in laboratory animals with implanted tumors provides data on the antitumor potential and the possibility of conducting clinical trials on oncotherapy in humans. Understanding of the processes of selective cytotoxic action, the mechanisms of the spread and cell tropism of the virus, its ability to affect angiogenesis in the tumor tissue as well as the immune system is important for the development of a prototype drug based on a wild-type NDV strain.

## 5. Conclusions

In conclusion, the intratumoral injections of the wild-type NDV/Altai/pigeon/770/2011 strain in BALB/c mice with intramuscular Krebs-2 carcinoma have a restraining effect on the tumor node growth. Furthermore, an additional mechanism suppressing tumor growth with NDV treatment is possibly regulation of the angiogenesis in tumor that leads to the spread of extensive necrotic foci. These results suggest that wild-type NDV strain has the antitumor properties to be able to successfully kill tumor and strains can serve as the perspective effective antitumor drug.

## Figures and Tables

**Table 1:** Volume density of necrosis and marker expression in Krebs-2 carcinoma during the experiment.

Group	Study Duration (Days)	(Vv) of Necrosis, %	ANLV	(Nai) CD34+ Cells	(Nai) VEGFR+ Cells
Control	5	8.8 ± 1.76	23.7	4.49 ± 0.48	14.92 ± 1.05
	10	13.5 ± 1.34	5.9	1.75 ± 0.22	19.92 ± 1.33
	15	15.3 ± 2.51	7.0	–	–
	20	19.8 ± 3.55	29.1	2.31 ± 0.30	10.44 ± 0.96
NDV-treated animals	5	16.7 ± 2.35 ^a^	12.4	2.60 ± 0.28 ^b^	15.51 ± 0.99
	10	15.5 ± 1.51	3.8	2.08 ± 0.24	11.88 ± 0.86 ^b^
	15	13.6 ± 1.95	9.2	–	–
	20	25.7 ± 2.68	5.9	0.98 ± 0.15 ^b^	6.53 ± 0.54 ^a^

^a^—*p* <0.01; ^b^—*p* < 0.001; significance of differences in the treated animals compared to the control, ANLV—average number of large vessels in the field of view (20× magnification). Changes in the volume density of necrosis in the tumor tissue are presented as relative mean values ± standard deviation.

## Data Availability

Not applicable.
